# A mini-open technique for Achilles tenotomy in infants with clubfoot

**DOI:** 10.1007/s11832-016-0710-3

**Published:** 2016-01-29

**Authors:** Rhett MacNeille, William Hennrikus, Brian Stapinski, Garrett Leonard

**Affiliations:** Department of Orthopaedics, Penn State Milton S. Hershey Medical Center, 30 Hope Drive, Entrance B, Suite 2200, Hershey, PA 17033 USA

**Keywords:** Ponseti, Clubfoot, Achilles tenotomy, Mini-open, TAL

## Abstract

**Purpose:**

A tendoachilles lengthening (TAL) is indicated in over 85 % of cases treated with the Ponseti technique. A percutaneous TAL is often performed in the clinic. Reported complications from a TAL performed in the clinic include: bleeding due to injury to the peroneal artery, posterior tibial artery, or lesser saphenous vein; injury to the tibial or sural nerves; and incomplete release. The purpose of the present study is to report the results and complications of a mini-open TAL performed in the operating room (OR).

**Methods:**

The current study is a retrospective review performed among infants with idiopathic clubfoot who underwent a mini-open TAL from 2008 to 2015.

**Results:**

Forty-one patients underwent 63 TALs via a mini-open technique in day surgery. The average Pirani score was 5.8 prior to casting. The average number of casts applied prior to surgery was 5.2. The average age at the time of the TAL was 12.5 weeks (range 5–48 weeks). The average weight at the time of surgery was 7.3 kg (range 3.6–13 kg). No child had a delay in discharge or stayed overnight in the hospital. No anesthesia-related complications or neurovascular injuries occurred. No child needed a repeat TAL due to an incomplete tenotomy.

**Conclusions:**

In conclusion, mini-open TAL performed in the OR is safe and effective in infants with clubfeet. No complications occurred and all patients were discharged on the day of surgery. Direct visualization of the Achilles tendon via a mini-open technique minimizes the risk of neurovascular injury and incomplete tenotomy.

## Introduction

Complications resulting from percutaneous Achilles tendon release (tendoachilles lengthening, TAL) performed in the clinic for the treatment of infants with clubfoot include: bleeding due to injury to the peroneal artery, posterior tibial artery, or lesser saphenous vein; injury to the tibial or sural nerves; and incomplete release [1–4]. Although Dr. Ponseti performed the TAL in the office under local anesthesia, some surgeons now perform the procedure in the operating room (OR) to optimize the child’s analgesia, improve safety, improve control of the procedure, and minimize complications [5, 6]. The purpose of this study is to report the outcomes of infants with clubfoot treated with a TAL in the OR via a mini-open approach.

## Materials and methods

**Fig. 1:**
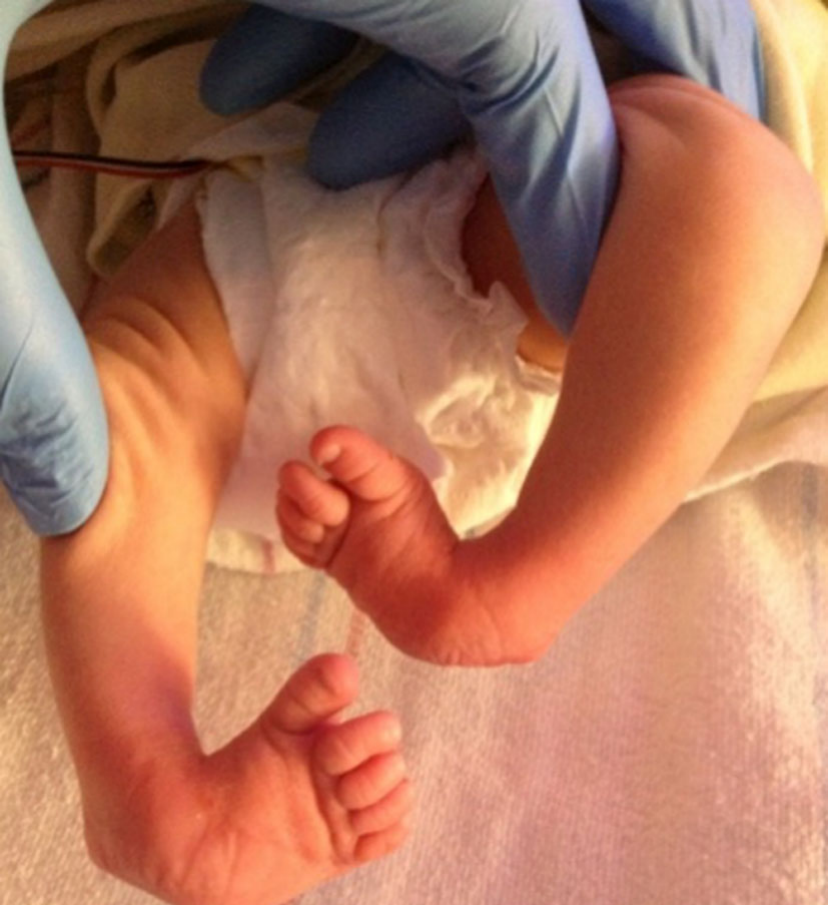
Bilateral clubfoot in a newborn child

**Fig. 2:**
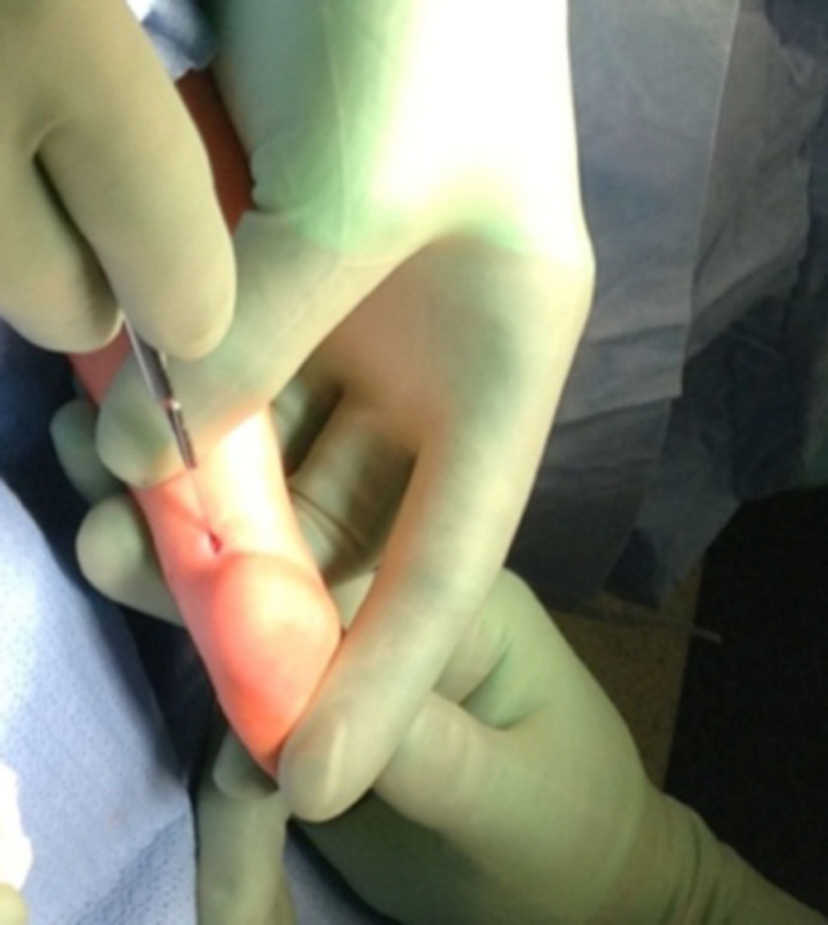
Small incision of the mini-open Achilles tenotomy. Incision made over the medial edge of the Achilles tendon

**Fig. 3:**
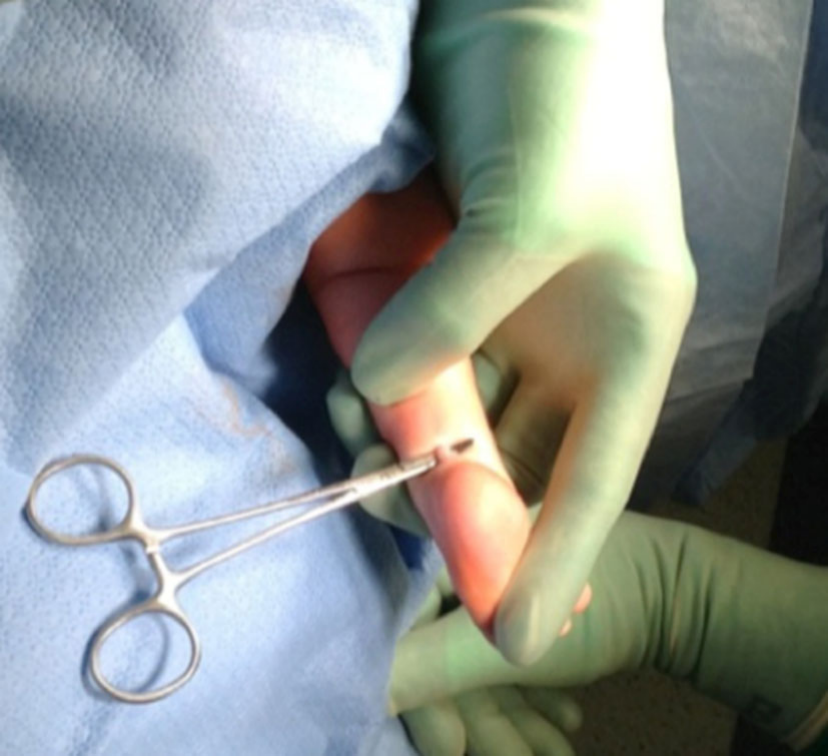
A small hemostat used to deliver the tendon from the wound for direct visualization prior to tenotomy

**Fig. 4:**
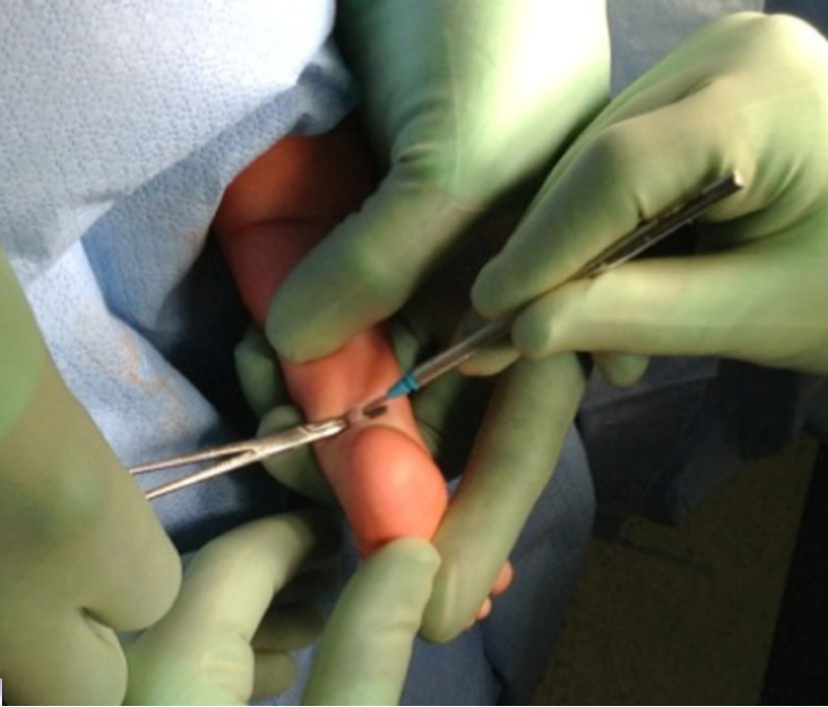
Tenotomy then performed only after direct visualization obtained

**Fig. 5:**
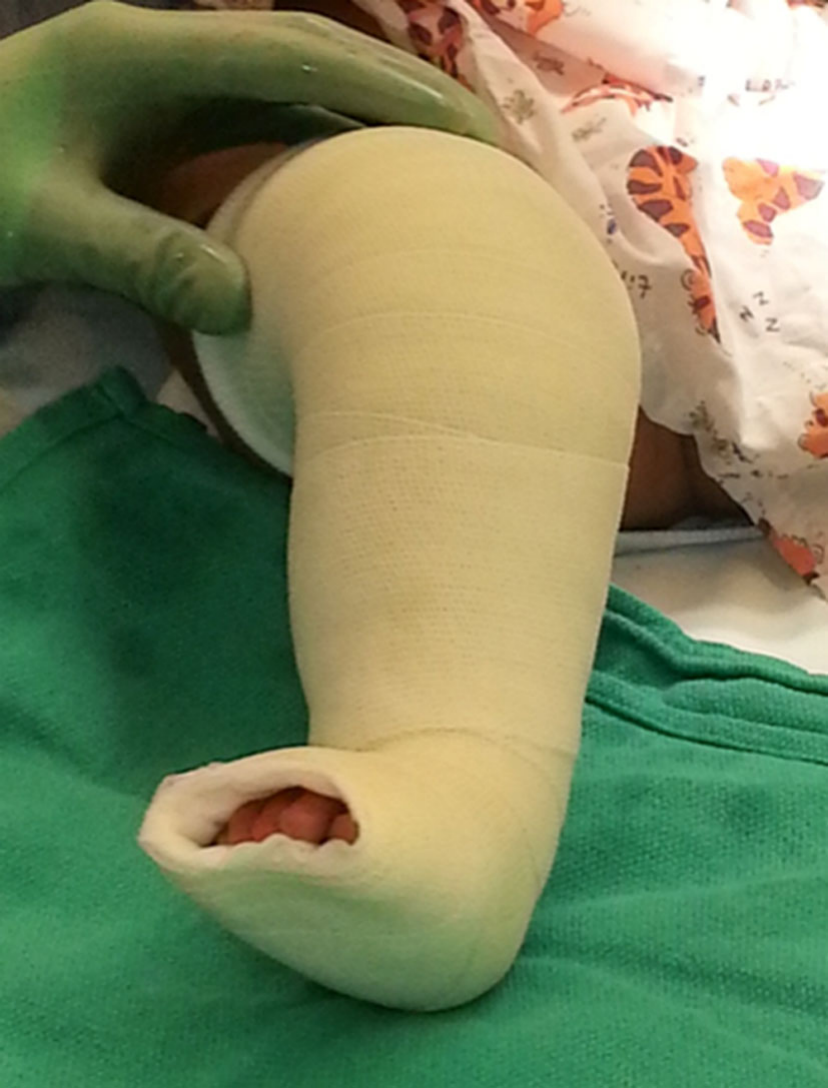
A long leg cast is applied for 4 weeks

A retrospective review was performed of infants with idiopathic clubfoot who underwent a mini-open TAL from 2008 to 2015. The data abstracted included: gender, number of casts, age at tenotomy, Pirani score, foot involvement, weight at surgery, anesthesia complications, need for admission, vascular or nerve injury, wound complications, and need for repeat surgery. All patients included in the present study had idiopathic clubfoot and excluded patients with any syndromic stigmata (Fig. 1). Patients undergoing TAL for toe walking or reasons other than clubfoot were excluded. A TAL was performed if the foot could not be dorsiflexed to 5° prior to application of the final cast. Sterile prep and drape was utilized. A time out was done. No tourniquet was utilized. The foot was maximally dorsiflexed and the distal portion of the Achilles tendon was palpated under the skin. A 10-mm incision was made along the medial edge of the tendon 1 cm above the calcaneus (Fig. 2). The Achilles tendon was identified. A mosquito clamp was place beneath the Achilles tendon from medial to lateral to isolate and tenotomize the tendon (Figs. 3 and 4). After tenotomy, an increase in dorsiflexion to greater than 5° was observed in all patients. Local anesthetic 2 ml of 0.25 % bupivacaine HCl with 1:200,000 epinephrine was injected. The skin was closed 4–0 Monocryl. A long leg cast was applied for 4 weeks (Fig. 5). One patient in the present study underwent a caudal block at the preference of the anesthesiologist. All other patients received general anesthesia and local anesthetic.

## Results

Forty-one patients underwent 63 TALs via a mini-open technique in day surgery. Twelve were right-sided, seven were left-sided, and 22 were bilateral TAL. The average Pirani score was 5.8 prior to casting (range 2–6). Pirani scoring consists of six components, each scored 0, 0.5, or 1, depending on severity, for a maximal score of 6 (Table 1). The average number of casts applied prior to surgery was 5.8, with a range of 3–10 casts applied. The average age at the time of the TAL was 12.5 weeks, with a range of 5–48 weeks. The average weight at the time of surgery was 7.3 kg, with a range of 3.6–13 kg. The average time in the day surgery unit was 5.2 h, with a range from 3 to 16 h (Table 2). No child had a delay in discharge or stayed overnight in the hospital. All children were prescribed acetaminophen upon discharge. No post-discharge phone calls were received from the families. No patient demonstrated an anesthetic-related complication, such as death, aspiration, arrhythmia, laryngeal spasm, or bronchospasm. No nerve injuries or vascular injuries occurred. No child needed a repeated TAL due to an incomplete tenotomy.

**Table 1:** Pirani score criteria. Pirani scoring consists of these six components, each scored 0, 0.5, or 1, depending on severity. The minimum total score is 0 and the maximum total score is 6

Pirani scoring	
1	Posterior crease
2	Empty heel
3	Rigid equinus
4	Medial crease
5	Curvature of the lateral border
6	Position of talar head

**Table 2:** Case data totals/averages with associated ranges for gender, Pirani score, laterality of clubfoot, number of casts, age (weeks), time spent in operating room (min), and time spent in hospital (min)

	Gender	Pirani score	Laterality	Number of casts	Age (weeks)	Operating room time (min)	Hospital time (min)
Total or average	M: 32 F: 9	5.8	R: 12 L: 7 B: 22	5.8	12.5	66.6	311.3
Range	−	2–6	−	3–10	5–48	48–111	180–960

## Discussion

The Ponseti technique involves weekly manipulations and serial casting to allow collagen relaxation and atraumatic remodeling of joint surfaces without fibrosis and scarring from surgical releases. The order of correction with serial casting progresses from addressing the midfoot cavus to forefoot adduction to hindfoot varus, then finally to hindfoot equinus [7]. If a residual equinus deformity is still present, a TAL is performed prior to the final cast application. The TAL is needed in about 85 % of cases [8].

Tendoachilles tenotomy in infants has been studied by ultrasound. The tendon reestablishes continuity between the stumps as soon as 3 weeks after surgery, with ability to transmit force to the heel [9–11]. Ultrasound was also used to measure calf diameters as a reflection of strength and showed no significant differences with the changes over time between affected and non-affected sides of children with unilateral clubfoot [12].

**Fig. 6:**
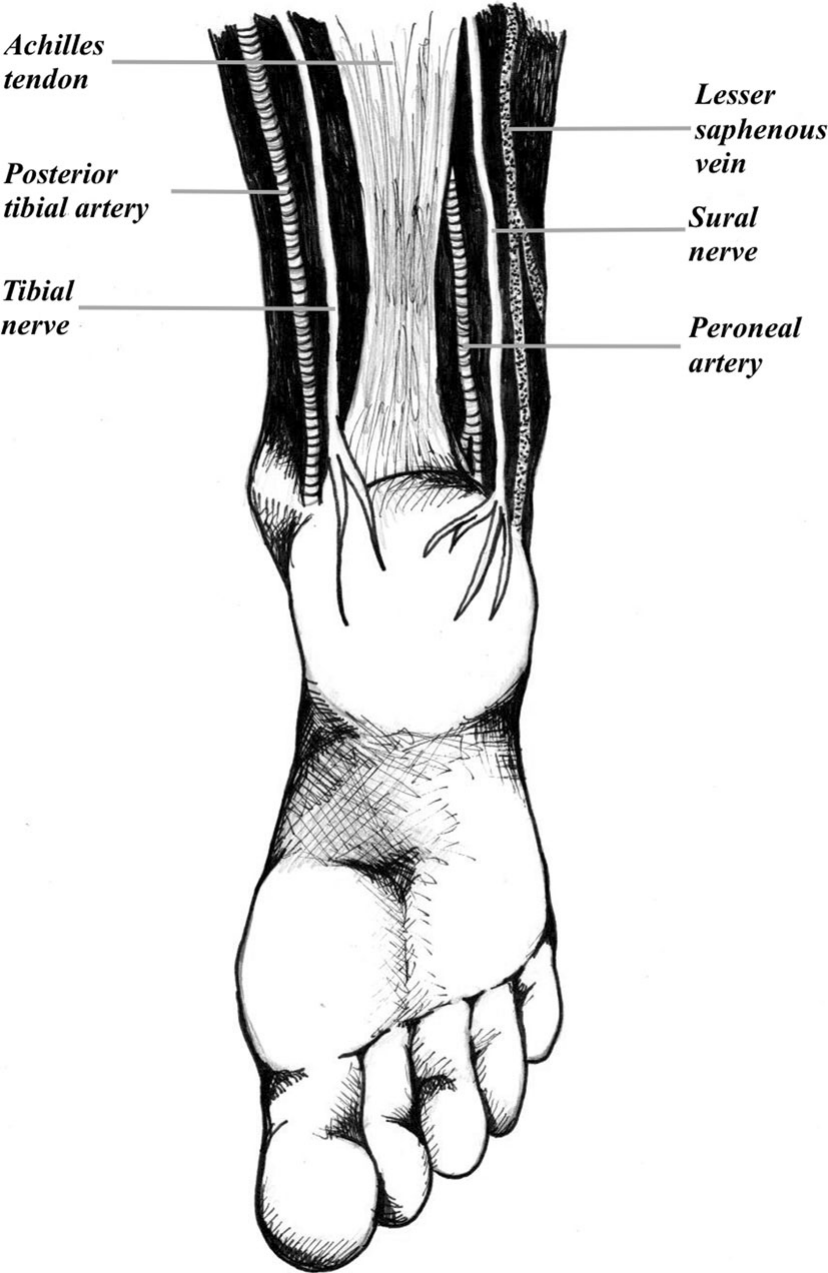
The anatomic structures at risk when performing a tendoachilles lengthening (TAL): peroneal and posterior tibial arteries; lesser saphenous vein; and tibial and sural nerves

Complications from percutaneous TAL in the clinic include bleeding due to injury to the peroneal artery, posterior tibial artery, or lesser saphenous vein, injury to the tibial or sural nerves, and incomplete release (Fig. 6) [1–4]. In an effort to avoid these complications, the senior author now performs a mini-open TAL as part of the Ponseti technique in the treatment of clubfoot. To the author’s knowledge, the mini-open technique has only been reported in the podiatric literature up to this point [13]. Anecdotal reports suggest a small open incision to directly visualize the tendon before tenotomy [3]. Dogan et al. corroborated their use of the mini-open technique with a rat study, demonstrating equivalent or better healing from a mini-open TAL compared with a percutaneous approach [14].

Ponseti and many clinicians have traditionally performed TAL in the clinic [8]. The benefits of performing the procedure in an outpatient office setting include decreased cost, no general anesthesia, and immediate treatment [15].

Other surgeons choose to perform the TAL in an OR setting [5, 16, 17]. The benefits include: improved safety, better control of the procedure for the surgeon, better ability to manage complications, and improved ability to safely guide a resident through the procedure. For example, in the present study, there were no adverse effects or complications from general anesthesia. Every patient was able to discharge to home the same day of the procedure. In addition, Iravani et al. demonstrated no adverse outcomes in 114 patients undergoing general anesthesia for TAL in clubfoot [5]. Bor et al. noted that too much use of local anesthetic can complicate the surgery by obscuring landmarks which may be especially problematic when performing percutaneous techniques [17]. In the current series, local anesthetic is applied after the tenotomy to avoid the local anesthetic obscuring surface landmarks or the surgical dissection.

The presence of vascular anomalies in the leg of patients with clubfoot is well documented. For example, in some cases, the anterior tibial artery and/or the posterior tibial artery can be absent [3]. In such feet, the peroneal artery is the dominant artery to supply the feet. Changulani et al. reported on a case study where the posterior artery was lacerated and the tibial nerve was completely transected during a percutaneous TAL. Injury to the peroneal artery in these patients may result in an avascular limb and a potential amputation [2]. Burghardt et al. also reported a case study on a child who developed a pseudoaneurysm after percutaneous TAL. Burghardt et al. noted that the patient had a posterior tibial artery-dependent foot. The child later required repeat tenotomy and, during the revision done by open tenotomy, the posterior tibial artery was noted to be closely applied to the Achilles tendon [1]. Dobbs et al. further highlighted this point, reporting on four patients with serious bleeding complications following percutaneous tendoachilles tenotomy due to injury to the peroneal artery in three of the cases and the lesser saphenous vein in one case [3]. In the current study, no case of vessel or nerve occurred when utilizing the mini open technique.

TAL in the OR is more costly than in the clinic [16]. Although the cost of performing the procedure in the outpatient clinic is less compared to in the OR, the cost of one complication, such as a preventable amputation in a newborn, would exceed the OR costs of performing TALs in the OR during one surgeon’s career. Anesthesia in small children is very safe but there are always risks. DiMaggio et al. reported that early exposure to child anesthesia resulted in increased risk of being diagnosed with behavioral and developmental disorders [18]. Wilder et al. also showed an increased risk with two or more early exposures, but a single exposure to anesthesia did not increase this risk [19]. No parent in this current study refused general anesthesia for his or her child.

TAL techniques range from percutaneous needle tenotomy to percutaneous tenotomy with a blade to open tenotomy to multiple tenotomies [3, 20–22]. A percutaneous technique with a blade or a needle may increase the chance of complications [13]. The percutaneous needle technique was described by Minkowitz et al. on 12 patients [20]. Another percutaneous technique was reported by Sharma et al. utilizing a mosquito hemostat to act as a depth gauge, helping to control the blade depth and as a backstop to protect the nearby neurovascular structures. In Sharma et al.’s technique, the Achilles tendon was still never visualized [23]. In the current study, direct visualization of the tendon via a mini-open technique minimizes risks and allows a complete tenotomy in all cases.

## Conclusion

Mini-open tendoachilles lengthening (TAL) in the operating room (OR) is safe and effective in infants with clubfeet. No complications occurred and all patients were discharged on the day of surgery. Direct visualization of the Achilles tendon via a mini-open technique minimizes the risk of neurovascular injury and incomplete tenotomy.
